# A new inbred strain of Fawn-Hooded rats demonstrates mania-like behavioural and monoaminergic abnormalities

**DOI:** 10.1016/j.ibror.2019.11.001

**Published:** 2019-11-06

**Authors:** Hirotsugu Azechi, Kōsuke Hakamada, Takanobu Yamamoto

**Affiliations:** a Department of Psychology, Tezukayama University, Nara 631-8585, Japan; b Department of Neurophysiology and Cognitive Neuroscience, Graduate School of Psychological Sciences, Tezukayama University, Nara 631-8585, Japan

**Keywords:** FH, Fawn-Hooded, 5-HT, serotonin, 5-HIAA, 5-hydroxyindoleacetic acid, HPLC, high-performance liquid chromatography, PCA, perchloric acid, NA, noradrenaline, DA, dopamine, SEM, standard error of the mean, MHPG, 3-methoxy-4-hydroxyphenylglycol, DOPAC, 3,4-dihydroxyphenylacetic acid, HVA, homovanillic acid, TPH2, tryptophan hydroxylase 2, MAO-A, monoamine oxidase A, ADHD, attention-deficit hyperactivity disorder, Fawn-Hooded rat, Hyperactivity, Impulsivity, Stimulus responsivity, Monoaminergic dysregulation, Bipolar mania model

## Abstract

The Fawn-Hooded (FH) rat carries a gene mutation that results in a dysfunctional serotoninergic system. However, previous studies have reported differing features between the FH/Wjd and FH/Har strains. We aimed to compare the behavioural and neurobiological features of FH/HamSlc rats with those of Fischer 344 rats. We performed the open field, elevated minus-maze, Y-maze spontaneous alternation, and forced swim tests to investigate behavioural alterations. We also assessed neurobiological characteristics by quantifying monoamines and their related compounds in the prefrontal cortex, hippocampus, and striatum using high-performance liquid chromatography with an electrochemical detection system. FH/HamSlc rats showed hyperactivity and a high impulsivity tendency in the open field and the elevated minus maze test, but no cognitive dysfunction. In addition, the hyperactivity was suppressed immediately after the forced swim test. FH/HamSlc rats showed low dopamine levels, but high dopamine turnover in the striatum. Serotonin and noradrenaline levels were low in the prefrontal cortex and the hippocampus of FH/HamSlc rats, but high serotonin turnover was observed in the prefrontal cortex, hippocampus, and striatum. FH/HamSlc rats show (1) mania-like behavioural characteristics that are different from those of other strains of FH rats; (2) stimulus dependent suppression of hyperactivity similar to the clinical findings that exercise alleviates the symptoms of bipolar disorder; and (3) monoaminergic dysregulation such as monoamine imbalance and hyperturnover that may be associated with mania-related behavioural characteristics. Thus, the FH/HamSlc rat is a new animal model for mania including bipolar disorder.

## 1 Introduction

Fawn-Hooded (FH) rats have a gene mutation in the recessive red-eyed dilution gene on chromosome 1, which is responsible for dysfunctional 5-hydroxytryptamine (5-HT) release from platelets (Tschopp and Zucker, 1972). In addition, hypertension and renal impairments have been attributed to *Bpfh-1*, and to *Rf-1* and *Rf-2*, respectively, in FH rats. These genes are close to the red-eyed dilution (Brown et al., 1996; Prieur and Meyers, 1984). In contrast to dysfunctional 5-HT release from platelets, FH rats express increased levels of 5-HT and its metabolite, 5-hydroxyindoleacetic acid (5-HIAA), in the brain and have altered responses to serotoninergic agents, indicating that FH rats have an altered serotoninergic system in the central nervous system (Gudelsky et al., 1985; Joseph, 1978). Therefore, FH rats are used as animal models for depression, but contrasting results have been reported regarding whether this strain can, in fact, model depression. Overstreet et al. reported that FH/Wjd rats show higher basal corticosterone levels and longer immobility times in the forced swim test, and that these abnormalities are normalised following antidepressant treatment, suggesting that the FH rat may be useful as an animal model of depression (Overstreet et al., 1992; Rezvani et al., 2002). Meanwhile, Lahmame et al. (1996) showed that FH/Har rats exhibit hyperactivity in some behavioural analyses and no change in corticosterone levels in response to tail-cut stress, indicating that the FH rat may not be an appropriate model of depressive disorder. It is likely that these inconsistencies are caused by differences in different FH strains (Overstreet and Rezvani, 1996). A direct comparison study between FH/Wjd and FH/Har rats has demonstrated that FH/Wjd rats exhibit longer immobility times than FH/Har rats in the forced swim test. However, FH/Har rats demonstrated increased anxiety-like behaviours in the elevated plus-maze test compared with FH/Wjd rats.

Clarifying the mechanisms underlying psychiatric disorders is critical for improving treatments. Animal models are useful to provide an understanding of the neurobiological mechanisms underlying these disorders and to enable the screening of therapeutic drugs (Takao and Miyakawa, 2015; Valvassori et al., 2013). Advancements in genetic engineering techniques have facilitated studying animals with mutations of candidate genes. However, in psychiatric disorders where it can be difficult to identify specific causal genes, animals with abnormal phenotypes, such as spontaneously mutated rats, are useful study tools. Primarily, animal models must have both face validity and constructive validity. Face validity refers to behavioural similarity with clinical symptoms, while constructive validity refers to biological similarity with disorders. If both validities are confirmed in an animal model, it can be used for determining the predictive validity. Predictive validity refers to whether a therapeutic drug is effective for treating behavioural and biological abnormalities. Ultimately, an animal model should establish these three validity types.

This study aimed to clarify the behavioural and neurobiological features of another FH strain, the FH/HamSlc rat, which is a descendent strain of the FH/Wjd and inbred strains created by Japan SLC Inc. Previous studies reported differences in features between strains; however, all FH rats are available for use as animal models of psychiatric disorders. To be relevant models of psychiatric disorders, these animals are required to exhibit specific behavioural and neurobiological abnormalities. Therefore, we evaluated the basic depression-, mania-, and anxiety-like behaviours in FH/HamSlc rats using a battery of behavioural tests. In addition, we determined the concentration of monoamines and their metabolites using high-performance liquid chromatography (HPLC). These analyses were compared with those of Fischer 344 rats, another inbred strain.

## 2 Materials and methods

### 2.1 Animals

Six male Fawn-Hooded (FH/HamSlc; Japan SLC, Inc.) and six male Fischer 344 rats (F344/NSlc; Japan SLC, Inc., hereafter referred to as F344) were used in this experiment. The ethical guidelines for animal experiments recommend the use of as few animals as possible. The number of animals used in this experiment was determined based on our previous studies (Yamamoto et al., 2012; Yamashita and Yamamoto, 2014). There are no established control rats that match the genetic background of FH rats; therefore, we used F344 rats for comparison to the FH rats because they are a genetically normal inbred strain. Two FH/HamSlc and two F344 rats were housed in each cage. Rats were allowed access to food and water ad libitum and housed in a room with controlled temperature (22 ± 1 °C) and humidity (55 %) on a 12-h light/dark cycle (lights on from 08:00 to 20:00). From 7 weeks of age, the rats were habituated to the experimenter via daily handling for 5 days (5 min/day per individual). All experiments were performed according to the guidelines established by the animal welfare committee of Tezukayama University (approval number: 2015-02).

### 2.2 Behavioural procedures

#### 2.2.1 Open field test

To evaluate locomotor activity and anxiety-related behaviour, we performed the open field test for 10 min. The apparatus was a circular field surrounded by a wall (diameter: 90 cm, wall height: 30 cm). Each rat was placed at the edge of the arena and allowed to explore freely. Rat behaviour was monitored using a CCD camera. The total distance travelled, movement duration, movement speed, and time spent in the centre arena (within a 60-cm diameter circle) were quantified using a CompACT VAS Video Tracking System for the Morris water maze Ver. 3.20 (MUROMACHI KIKAI Co., Ltd., Japan). The total distance travelled, movement duration, and movement speed were used as indices of locomotor activity, and time spent in the open arena was used as an index of anxiety.

#### 2.2.2 Elevated minus-maze test

To evaluate anxiety-related behaviour, we performed an elevated minus-maze test for 10 min, which is a simplified version of the elevated plus-maze test (Hakamada and Yamamoto, 2014). The apparatus consisted of an open arm (60 cm long ×15 cm wide) and a closed arm (60 cm long ×15 cm wide ×40 cm high). The arms were aligned in a straight line and were elevated to a height of 50 cm above the floor. Each rat was placed in the centre of the apparatus. Rat behaviour was recorded using a web camera (UCAM-DLY300TABK; ELECOM Co., Ltd., Japan) and a video editor (WebCam ASSISTANT ver 1.12.000; ELECOM Co., Ltd.). Rats were classified to be in either arm when their trunk was entirely within that arm. Time spent in the open arm was used as an index of anxiety.

#### 2.2.3 Y-maze spontaneous alternation test

To evaluate cognitive function, we assessed the spontaneous alternation of rats for 20 min using the Y-maze. This task is often used to evaluate cognitive function in animal model studies of psychiatric disorders (Amodeo et al., 2017; Nguyen et al., 2014; Suryavanshi et al., 2014) because it requires intact spatial working memory. The apparatus was made from a white plastic board (each arm was 60 cm long ×15 cm wide ×30 cm high). Each rat was placed in the centre of the apparatus and allowed to explore the three arms. Rat behaviours were recorded using a web camera and video editor, as described above. We recorded the sequence of arm entries. We defined a rat’s forelimb crossing the arm entrance as an arm entry or exit. If a rat entered all three arms in a consecutive triad (e.g., ABC, CAB, BAC), this was defined as alternation behaviour. The alternation ratio is the number of alternation behaviours divided by the number of maximum possible alternation behaviours (equivalent to the total number of arm entries minus two) and multiplied by 100. The alternation ratio was used as an index of spatial working memory, with a reduction in the alternation ratio indicating a deficit in spatial working memory.

#### 2.2.4 Forced swim and open field tests

To evaluate depression-like behaviour, we performed the forced swim test. The apparatus was a circular pool (147 cm diameter ×40 cm height) filled with water to a depth of 30 cm. Each rat was placed in the pool for 10 min. Recordings were acquired using a CCD camera and movement duration was recorded using the Video Tracking System described above. The immobility time was defined as ‘600 – movement duration (seconds).’After the forced swim test, the rats were dried with a towel and placed in the open field apparatus. Distance travelled and time spent in the centre arena (within a 60-cm diameter circle) were recorded for 60 min. Furthermore, 10 days after this test, which is considered the time for recovery from fatigue in the forced swim test, the rats were placed in the open field apparatus again for 60 min, in the absence of the forced swim test. These results were used as an index of stimulus responsiveness.

### 2.3 Neurochemical analysis

#### 2.3.1 Sample extraction from the brain

After all behavioural procedures had been performed, the prefrontal cortex, hippocampus, and striatum of the rats were excised at the age of 12 weeks. Tissue weight was measured, followed by homogenisation in perchloric acid (PCA) buffer for deproteinization (3 % PCA containing 1 mM Na_2_S_2_O_5_ and 0.2 % EDTA-2Na; volume = 5 × tissue weight). The homogenate was centrifuged at 10,000 *g* for 15 min, and the supernatant was extracted and stored at −80 °C for later HPLC analysis.

#### 2.3.2 Plasma isolation from blood

Whole blood was collected to isolate the plasma when the rats were euthanised. Next, 1 mg EDTA-2Na was added to 1 mL of the collected blood and centrifuged at 1000 *g* for 10 min, and then the plasma was extracted. Next, 3 % PCA (0.5 × plasma volume) was added, and the sample was centrifuged at 10,000 *g* for 15 min. The plasma sample was stored at −80 °C for later HPLC analysis.

#### 2.3.3 HPLC analysis

5-HT, noradrenaline (NA), dopamine (DA), and their related metabolites or compounds were determined using HPLC with an electrochemical detector system (HPLC-ECD; Shiseido Co., Ltd., Japan) and the C-R8A Chromatopac (Shimadzu Corporation, Japan). The analytical conditions were based on those of a previous study (Yamamoto et al., 1997) and were adjusted for the current study. The mobile phase was 10 % (v/v) methanol in a solution (pH 4.70) containing (in mM): 32 citric acid, 12.5 Na_2_HPO_4_, 0.01 sodium octyl sulphate, 50 NaCl, and 0.05 EDTA. The mobile phase was filtered through a 0.2-μm filter (Advantec, Toyo, Japan). We coupled the two analytical ODS columns (CAPCELLPAK C18 MG, reversed-phase 5 μm, 4.6 mm I.D., 150 mm; Shiseido Co., Ltd.) and maintained their temperature at 45 °C. A glass-carbon working electrode was set at 0.8 V (vs. Ag/AgCl), and a flow rate of 0.6 mL/min was employed. Supernatants and plasma were injected into the HPLC system.

### 2.4 Data analysis

Plasma compound concentrations are presented as nmol/mL. Brain monoamine and metabolite concentrations are presented as nmol/g wet tissue. Undetected compounds are presented as ND (no data). Each monoamine turnover was calculated as the monoamine metabolite divided by its parent monoamine [i.e. 5-HT turnover = 5-HIAA/5-HT, DA turnover = (DOPAC + HVA)/DA, and NA turnover = MHPG/NA]. All data are presented as the mean ± standard error of the mean (SEM). Comparisons between rat strains were performed using t-tests, and comparisons of multiple factors were performed using two-way analyses of variance (ANOVAs). Differences were deemed statistically significant if p < 0.05. All statistical analyses were carried out using IBM SPSS Statistics ver. 21 (Japan IBM, Japan).

## 3 Results

### 3.1 Behavioural tests

#### 3.1.1 Open field test

**Fig. 1:**
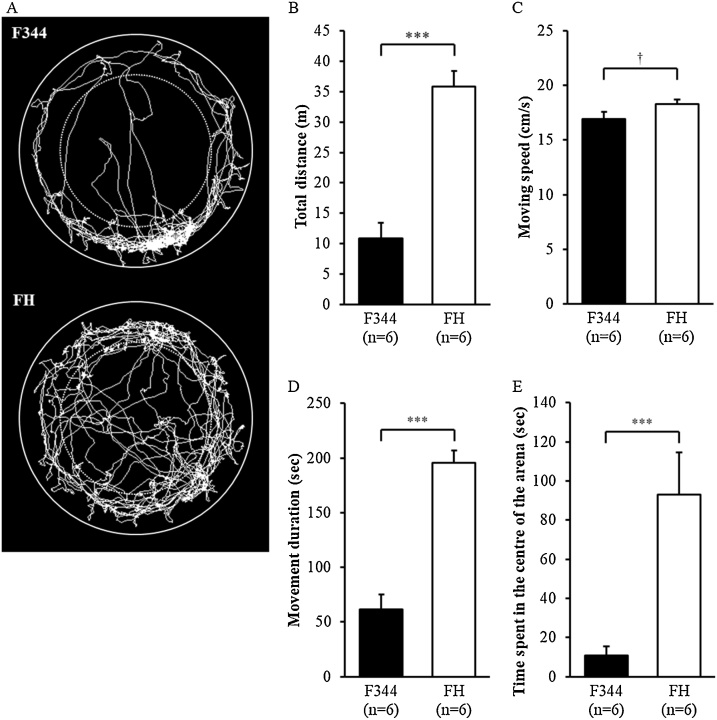
Locomotor activity and anxiety-related behaviour in the open field test. A: Representative traces of rat behaviour during a 10-min period (upper: F344, lower: FH/HamSlc). The inner thin circles denote the centre areas. B–E: Results are shown for total distance travelled (B), movement speed (C), movement duration (D), and time spent in the centre of the arena (E). Data are presented as the mean and SEM. F344, Fischer 344 rats (n = 6); FH, Fawn-Hooded/HamSlc rats (n = 6). Asterisks indicate a statistically significant difference (***p < 0.001); ^†^p < 0.10.

Representative traces recorded during the open field test are presented in Fig. 1A. FH/HamSlc rats showed significantly longer distances travelled (Fig. 1B; t(10) = 6.873, p < 0.001) and movement durations (Fig. 1D; t(10) = 7.593, p < 0.001) compared with the F344 rats. Movement speeds were numerically but not significantly faster in the FH/HamSlc rats (Fig. 1C; t(10) = 1.910, p = 0.085). The FH/HamSlc rats spent more time in the centre of the arena compared with the F344 rats (Fig. 1E; t(10) = 3.729, p < 0.001).

#### 3.1.2 Elevated minus-maze test

**Fig. 2:**
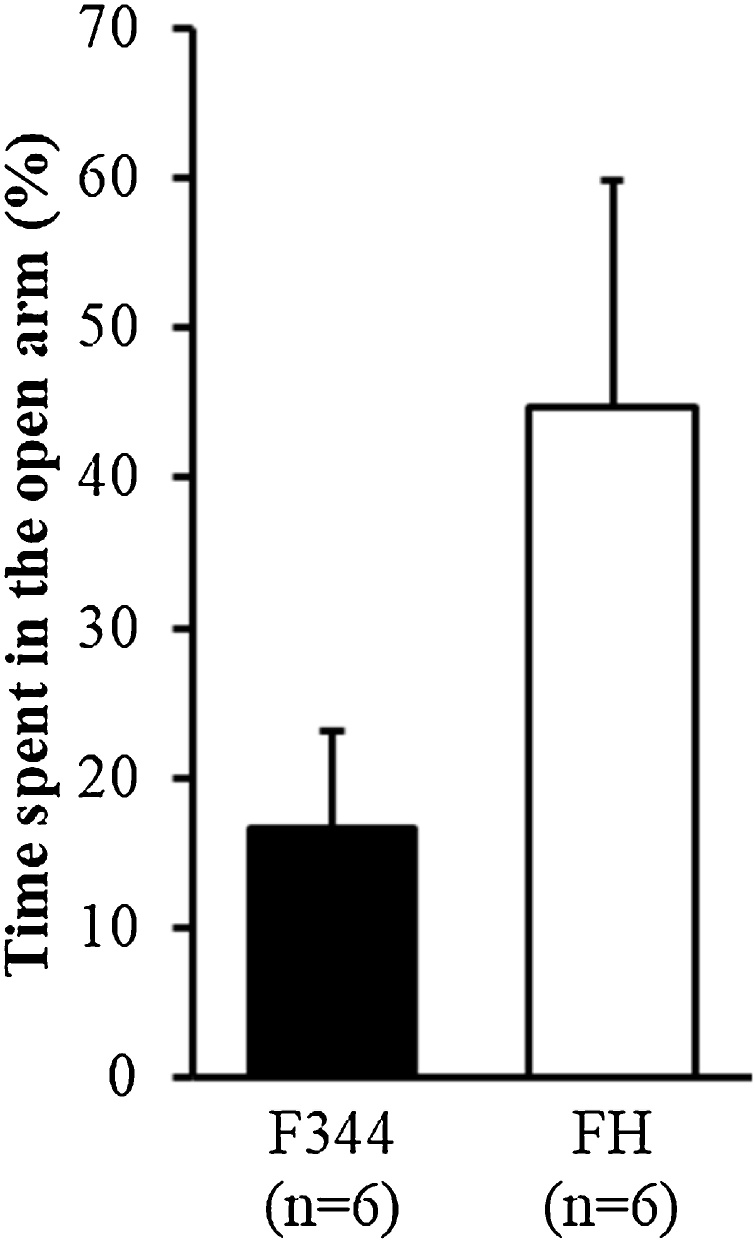
Anxiety-related behaviour in the elevated minus-maze test. The percentage of time spent in the open arm during a 10-min period. Data are presented as the mean and SEM. F344, Fischer 344 rats (n = 6); FH, Fawn-Hooded/HamSlc rats (n = 6).

The duration spent in the open arm was four times longer in the FH/HamSlc rats than in the F344 rats; however, this did not reach statistical significance (Fig. 2; t(6.8) = 1.699, p = 0.135).

#### 3.1.3 Spontaneous alternation test

**Fig. 3:**
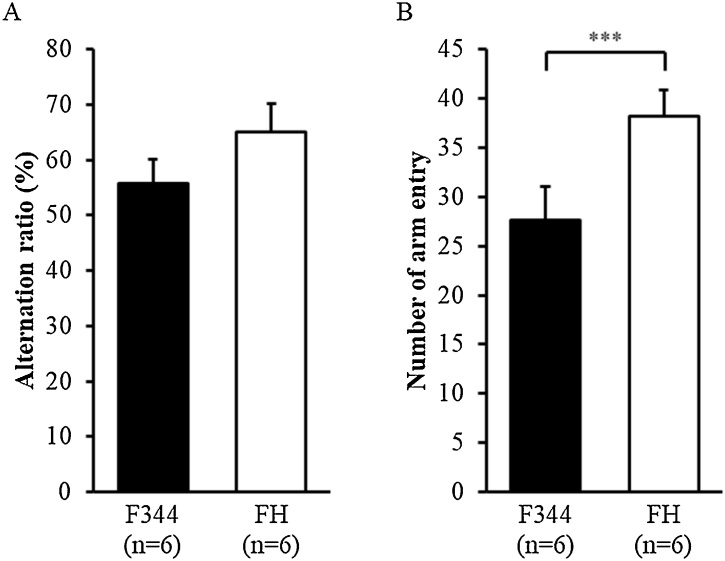
Short-term memory performance in the spontaneous alternation test. A, B: Results of the alternation ratio (A) and total number of arm entries (B) over a 20-min period. Data are presented as the mean and SEM. F344, Fischer 344 rats (n = 6); FH, Fawn-Hooded/HamSlc rats (n = 6). Asterisks indicate a statistically significant difference (***p < 0.001).

There was no significant difference in the alternation ratio between the FH/HamSlc and F344 rats (Fig. 3A; t(10) = 1.379, p = 0.198). However, the number of total arm entries was significantly higher in the FH/HamSlc rats than in the F344 rats (Fig. 3B; t(10) = 2.426, p = 0.036).

#### 3.1.4 Forced swim and open field tests

**Fig. 4:**
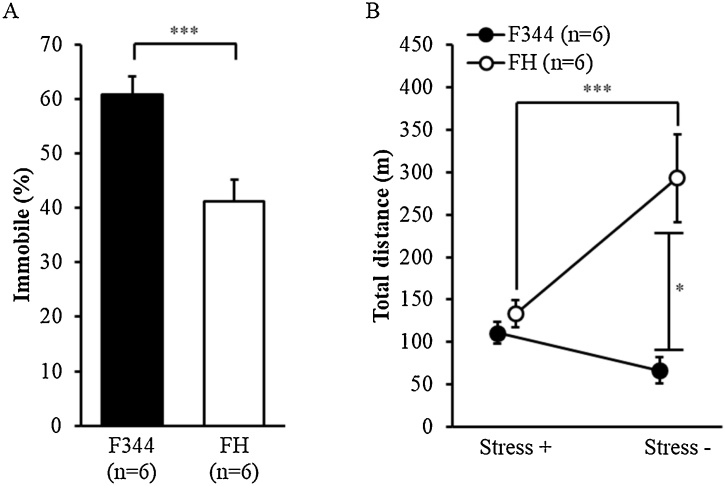
Depression-related behaviours in the forced swim test and the following open field test. A: Immobility time during the forced swim test within a 10-min period. B: Total distance travelled in the open field test over a 60-min period immediately after or without a prior forced swim test. Data are presented as the mean and SEM. F344, Fischer 344 rats (n = 6); FH, Fawn-Hooded/HamSlc rats (n = 6). Asterisks indicate a statistically significant difference (***p < 0.001, *p < 0.05).

The FH/HamSlc rats showed significantly shorter times spent immobile than the F344 rats in the forced swim test (Fig. 4A; t(10) = 3.718, p = 0.004). We further analysed the stressor factors and distance travelled in both rat strains in the open field test using the two-way ANOVA. There was a significant interaction between the forced swim test stressor and strain (Fig. 4B; F(1, 10) = 14.170, p = 0.004). Post hoc analyses revealed that there was no significant difference in the distance travelled in the presence of the forced swim stressor between the FH/HamSlc and F344 rats (p = 0.371); however, the FH/HamSlc rats showed a significantly longer distance travelled in the absence of the stressor compared with the F344 rats (p < 0.001). There was no difference in the distance travelled in the presence or absence of the forced swim stressor in the F344 rats (p = 0.885).

### 3.2 Body and brain weight

The results of the body and brain weight assessment, as well as the brain-to-body weight ratio, are shown in Table 1. Body weight was significantly higher in the FH/HamSlc rats than in the F344 rats at 8, 10, and 12 weeks (8 weeks: t(10) = 7.231, p < 0.001; 10 weeks: t(10) = 6.383, p < 0.001; 12 weeks: t(10) = 7.598, p < 0.001), but not at 4 weeks (t(6.6) = 2.119, p = 0.074). While the mean body weight was significantly higher in the FH/HamSlc rats than in the F344 rats, the mean brain weight of the FH/HamSlc rats was significantly lower (t(10) = 9.422, p < 0.001). Therefore, the brain-to-body weight ratio was reduced in the FH rats in comparison to the F344 rats (t(10) = 11.580, p < 0.001).

**Table 1:** Developmental changes in body and brain weights in F344 and FH/HamSlc rats.

	4 weeks Body weight (g)	8 weeks Body weight (g)	10 weeks Body weight (g)	12 weeks Body weight (g)	12 weeks Brain weight (g)	12 weeks Brain/body (%)
F344 (n = 6)	67.83 ± 1.14	187.00 ± 3.14	226.67 ± 4.63	262.50 ± 5.30	1.88 ± 0.01	0.72 ± 0.01
FH/HamSlc (n = 6)	74.33 ± 2.85^†^	233.17 ± 5.56***	273.67 ± 5.73***	321.50 ± 5.67***	1.71 ± 0.02***	0.53 ± 0.01***

Mean ± SEM; ^†^p < 0.10, ***p < 0.001.

F344, Fischer 344; FH, Fawn-Hooded.

### 3.3 Concentrations of tryptophan, monoamines, and monoamine metabolites

#### 3.3.1 Plasma

The plasma concentrations of 5-HT and tryptophan are shown in Table 2. FH rats exhibit dysfunctional 5-HT release from platelets; therefore, their plasma 5-HT concentration is low (Tschopp and Zucker, 1972). In concordance with this, we found that the plasma 5-HT concentration was significantly lower in the FH rats than in the F344 rats (t(5.1) = 9.424, p < 0.001). Moreover, the plasma concentration of tryptophan was reduced in the FH/HamSlc rats compared with that in the F344 rats (t(9) = 2.287, p = 0.048).

**Table 2:** Plasma 5-HT and tryptophan concentrations in F344 and FH/HamSlc rats.

	5-HT	Tryptophan
F344 (n = 6)	4.41 ± 0.36	59.49 ± 4.74
FH/HamSlc (n = 5)	0.98 ± 0.04***	46.05 ± 2.96*

Mean ± SEM (nmol/mL); *p < 0.05, ***p < 0.001.

5-HT, 5-hydroxytryptamine (serotonin); F344, Fischer 344; FH, Fawn-Hooded.

#### 3.3.2 Brain

Table 3, Table 4 show the concentrations of brain monoamines and their metabolites, as well as the monoamine turnover. In the prefrontal cortex, the concentrations of 5-HT and NA were reduced in the FH/HamSlc rats compared with those in the F344 rats, while there was no significant difference in the DA concentration between the FH/HamSlc and F344 rats (5-HT: t(10) = 5.645, p < 0.001; NA: t(10) = 2.252, p = 0.048; DA: t(6.5) = 2.281, p = 0.059). Furthermore, there were no significant differences between the FH/HamSlc and F344 rats in the concentration of the metabolites, 5-HIAA, 3-methoxy-4-hydroxyphenylglycol (MHPG), 3,4-dihydroxyphenylacetic acid (DOPAC), and homovanillic acid (HVA) (5-HIAA: t(6.5) = 0.647, p = 0.540; MHPG: t(6.9) = 0.768, p = 0.267; DOPAC: t(5.2) = 0.254, p = 0.809; HVA: t(5.0) = 0.579, p = 0.588). By contrast, the tryptophan concentration in the FH/HamSlc rats was significantly increased compared with that in the F344 rats (t(10) = 3.468, p = 0.006). Additionally, the 5-HT turnover was enhanced in the FH/HamSlc rats compared with that in the F344 rats, but there was no difference in NA or DA turnover (5-HT turnover: t(10) = 3.775, p = 0.004; NA turnover: t(10) = 0.375, p = 0.715; DA turnover: t(10) = 1.576, p = 0.146).

**Table 3:** Concentrations of brain monoamines and their metabolites in the brains of F344 and FH/HamSlc rats.

	5-HT	5-HIAA	Tryptophan	NA	MHPG	DA	DOPAC	HVA
Prefrontal cortex								
F344 (n = 6)	0.99 ± 0.04	0.44 ± 0.04	9.61 ± 0.50	1.05 ± 0.06	0.09 ± 0.02	0.30 ± 0.05	0.29 ± 0.04	0.17 ± 0.08
FH/HamSlc (n = 6)	0.71 ± 0.02***	0.47 ± 0.02	11.64 ± 0.30**	0.91 ± 0.02*	0.06 ± 0.01	0.18 ± 0.02^†^	0.28 ± 0.01	0.12 ± 0.01
Hippocampus								
F344 (n = 6)	0.85 ± 0.03	0.70 ± 0.03	9.77 ± 0.47	1.76 ± 0.04	0.02 ± 0.01	0.04 ± 0.01	0.31 ± 0.02	ND
FH/HamSlc (n = 6)	0.68 ± 0.04**	0.83 ± 0.06^†^	10.18 ± 0.29	1.18 ± 0.06***	0.05 ± 0.03	0.03 ± 0.01	0.27 ± 0.01	ND
Striatum								
F344 (n = 6)	0.82 ± 0.04	0.76 ± 0.03	9.34 ± 0.49	0.36 ± 0.06	ND	33.23 ± 1.15	4.09 ± 0.23	2.36 ± 0.21
FH/HamSlc (n = 6)	0.77 ± 0.04	0.85 ± 0.04	9.66 ± 0.30	0.43 ± 0.04	ND	27.61 ± 1.18**	4.55 ± 0.23	3.39 ± 0.15**

Mean ± SEM (nmol/g wet tissue); ^†^p < 0.10, *p < 0.05, **p < 0.01, ***p < 0.001.

F344, Fischer 344; FH, Fawn-Hooded; 5-HT, 5-hydroxytryptamine (serotonin); 5-HIAA, 5-hydroxyindoleacetic acid; NA, noradrenaline; MHPG, 3-methoxy-4-hydroxyphenylglycol; DA, dopamine; DOPAC, 3,4-dihydroxyphenylacetic acid; HVA, homovanillic acid; ND, no data (undetected in HPLC).

**Table 4:** 5-HT, NA, and DA turnovers in F344 and FH/HamSlc rats.

		5-HT turnover (5-HIAA/5-HT)	NA turnover (MHPG/NA)	DA turnover ([DOPAC + HVA]/DA)
Prefrontal cortex	F344 (n = 6)	0.445 ± 0.052	0.078 ± 0.016	1.591 ± 0.419
	FH/HamSlc (n = 6)	0.661 ± 0.025**	0.060 ± 0.009	2.348 ± 0.235
Hippocampus	F344 (n = 6)	0.836 ± 0.038	0.011 ± 0.002	ND
	FH/HamSlc (n = 6)	1.222 ± 0.048***	0.043 ± 0.029	ND
Striatum	F344 (n = 6)	0.938 ± 0.194	ND	0.194 ± 0.011
	FH/HamSlc (n = 6)	1.108 ± 0.040**	ND	0.288 ± 0.004***

Mean ± SEM; **p < 0.01, ***p < 0.001.

5-HT, 5-hydroxytryptamine (serotonin); NA, noradrenaline; DA, dopamine; F344, Fischer 344; FH, Fawn-Hooded; 5-HIAA, 5-hydroxyindoleacetic acid; MHPG, 3-methoxy-4-hydroxyphenylglycol; DOPAC, 3,4-dihydroxyphenylacetic acid; HVA, homovanillic acid; ND, no data.

In the hippocampus, the concentrations of 5-HT and NA were reduced in the FH/HamSlc rats, while the 5-HIAA concentration was increased, compared with the concentrations in the F344 rats (5-HT: t(10) = 3.239, p = 0.009; NA: t(10) = 8.174, p < 0.001; 5-HIAA: t(10) = 1.881, p = 0.089). The 5-HT turnover was enhanced in the FH/HamSlc rats in comparison to the F344 rats (t(10) = 6.335, p < 0.001). There were no differences in tryptophan, MHPG, DA, DOPAC, or NA turnover between the FH/HamSlc and F344 rats (tryptophan: t(10) = 0.755, p = 0.468; MHPG: t(5.2) = 0.857, p = 0.429; DA: t(10) = 1.160, p = 0.273; DOPAC: t(10) = 1.651, p = 0.130; NA turnover: t(5.1) = 1.068, p = 0.334).

In the striatum, the DA concentration was reduced and the HVA concentration was increased in the FH/HamSlc rats compared with those in the F344 rats (DA: t(10) = 3.409, p = 0.007; HVA: t(10) = 3.933, p = 0.003). There were no differences in the concentrations of the other assessed compounds between the FH/HamSlc and F344 rats (5-HT: t(10) = 0.939, p = 0.370; tryptophan: t(10) = 0.560, p = 0.588; 5-HIAA: t(10) = 1.615, p = 0.137; NA: t(10) = 1.019, p = 0.332; DOPAC: t(10) = 1.367, p = 0.202). Both 5-HT and DA turnover were enhanced in the FH/HamSlc rats (5-HT turnover: t(10) = 3.605, p = 0.005; DA turnover: t(10) = 8.187, p < 0.001).

## 4 Discussion

In the present study, we investigated whether FH/HamSlc rats show behavioural and neurobiological abnormalities. There have been contrasting findings across different FH strains: while some studies have suggested that the FH/Wjd strain can be used as a model of depression, other studies have reported that the FH/Har strain does not show behaviours associated with a model of depression (Lahmame et al., 1996; Overstreet and Rezvani, 1996; Overstreet et al., 1992; Rezvani et al., 2002). Our results show that the FH/HamSlc strain exhibits mania-like traits in behavioural and neurobiological analyses, which indicates that it could be used as a model for mania including bipolar disorder.

Our behavioural analysis revealed that FH/HamSlc rats are more hyperactive in the open field (Fig. 1B), spontaneous alternation (Fig. 3B), and forced swim (Fig. 4A) tests. This hyperactivity, which may be attributed to psychomotor excitation, resulted mainly from increases in movement duration, rather than in movement speed (Figs. 1C and D). Although time spent in the open arm was four times higher in the FH/HamSlc rats than in the F344 rats, there was no significant change in anxiety-related behaviours in the elevated minus-maze test (Fig. 2). Nevertheless, the FH/HamSlc rats spent more time in the centre of the open field than the F344 rats (Fig. 1E), which suggests low anxious tendencies in FH/HamSlc rats. Attention-deficit hyperactivity disorder (ADHD) animal models have been analysed using access to the open arm of the elevated plus maze as an indicator of impulsivity (Hiraide et al., 2013; Hakamada and Yamamoto, 2014), because there is a negative correlation between impulsivity and anxiety level in children with ADHD (Pliszka, 1989). These low-anxiety behaviours observed in our FH/HamSlc rats may reflect high impulsivity. Furthermore, in human, high impulsivity is associated with taking risky behaviours (Donohew et al., 2000; Bakhshani, 2014). These behavioural features in the FH/HamSlc rats appear to mimic mania rather than depression and anxiety disorder. However, the hyperactivity in the FH/HamSlc rats was transiently suppressed following stress loading using the forced swim test (Fig. 4B). This behavioural suppression is not due to habituation to the environment, because the rats exhibited hyperactivity in subsequent open field tests performed under non-stress conditions. No behavioural suppression was observed in the F344 rats, indicating that FH/HamSlc rats respond differently to stress. This stimulus-dependent behavioural suppression appears to be similar to the finding that physical exercise improves symptoms of bipolar disorder (de Sá Filho et al., 2015; Vancampfort et al., 2017). In this study, although we used the forced swim test for distress loading, this task may in fact have induced eustress. Although mania often causes cognitive dysfunction, the FH/HamSlc rats did not show any significant change in cognitive function, as assessed by the spontaneous alternation test. These results indicate that FH/HamSlc rats have behavioural features related to mania without clear cognitive dysfunction.

Monoaminergic functions have been shown to be related to manic and depressive states. FH rats have a spontaneous mutation of the recessive red-eyed dilution gene (also known as *Rab38*) on chromosome 1 (Prieur and Meyers, 1984) and exhibit dysfunctional 5-HT release from platelets, as well as serotoninergic system alterations in the central nervous system (Gudelsky et al., 1985; Joseph, 1978; Tschopp and Zucker, 1972). Consistent with previous studies (Joseph, 1978), we found a reduction in plasma 5-HT levels in the FH/HamSlc rats compared with the F344 rats (Table 2). Furthermore, the concentration of tryptophan was low in the plasma but high in the brain, particularly in the prefrontal cortex (Table 2, Table 3). Tryptophan, an essential amino acid, cannot permeate the blood-brain barrier and is delivered to the brain from the periphery via l-type amino acid transporter 1 (Boado et al., 1999; Pardridge, 1979). However, blood-brain barrier permeability is increased by stress (Esposito et al., 2002, 2001). Therefore, we presume that since FH/HamSlc rats exhibit high responsivity to stimuli (Fig. 4B), they might experience an increased influx of tryptophan into the brain and a subsequent decrease in peripheral tryptophan, even under mild stress.

An increase in brain tryptophan levels could enhance 5-HT synthesis in the central nervous system; however, in this study, the FH/HamSlc rats had reduced 5-HT levels in the prefrontal cortex and hippocampus (Table 3). In the prefrontal cortex, the FH/HamSlc rats showed a high concentration of tryptophan and a low concentration of 5-HT (Table 3). Conversely, the FH/HamSlc rats showed a low concentration of 5-HT but a high concentration of 5-HIAA in the hippocampus (Table 3). In contrast to the hippocampal results, we did not find any changes in the concentration of 5-HIAA in the prefrontal cortex and striatum (Table 3). Nevertheless, 5-HT turnover was enhanced in both of these brain regions, as well as in the hippocampus (Table 4). Degradation of 5-HT was also enhanced relative to the 5-HT concentration in the prefrontal cortex and striatum of the FH/HamSlc rats, compared with the F344 rats. These results suggest that the regulatory mechanism of the 5-HT metabolic pathway is altered in FH/HamSlc rats, probably through enzyme activity. A low 5-HT concentration would be caused by suppression of tryptophan hydroxylase 2 (TPH2) in the prefrontal cortex and hypofunction of monoamine oxidase A (MAO-A) in the hippocampus. TPH2 is a rate-limiting enzyme of the 5-HT metabolic pathway in the brain that catalyses the conversion of tryptophan to 5-hydroxytryptophan. Therefore, a decreased function of this enzyme induces an elevation of tryptophan levels. MAO-A catalyses the conversion of 5-HT to 5-HIAA in the 5-HT metabolic pathway in the brain. Therefore, we presume that TPH2 hypofunction and MAO-A hyperfunction may induce the change in the regulatory mechanism of the 5-HT metabolic pathway observed in the FH/HamSlc rats. Interestingly, a *TPH2* gene polymorphism or TPH2 activation has been reported in patients with depressive and bipolar disorder (Cichon et al., 2008; Fukuda, 2014). In addition, alterations in MAO-A expression or function have been shown to affect 5-HT degradation and behaviour (Kabayama et al., 2013).

Clinical studies have reported that the D2 and D3 receptor partial agonist cariprazine can affect manic symptoms (Altınbaş et al., 2013), and biomarker studies have reported that HVA is increased in the cerebrospinal fluid of patients with bipolar disorder (Pålsson et al., 2017). Thus, the dopaminergic system is thought to contribute to manic symptoms. In this study, the FH/HamSlc rats showed significantly higher HVA and/or DA turnover than the F344 rats in the striatum (Table 3, Table 4). In this study, we did not analyse the dorsal and ventral striatum separately. We extracted both the dorsal and ventral striatum together. The dorsal striatum (also known as the neostriatum) is composed of the putamen and caudate nucleus, which participate in the cortico-basal ganglia loop to regulate motor functions by nigrostriatal DA transmission. Therefore, dysregulation of this transmission can cause behavioural hyper- or hypoactivity. Low striatal DA or high DA turnover in FH/HamSlc rats may be associated with behavioural hyperactivity under the non-stress basal condition. By contrast, the ventral striatum, which includes the tuberculum olfactorium and nucleus accumbens, receives projections from the ventral tegmental area (mesolimbic dopaminergic, or reward, system) and is related to the generation of positive moods, such as pleasure; a decline in the function of this pathway is associated with a negative mood in depressive patients. However, in this study, FH/HamSlc rats did not appear to show behavioural changes associated with a depressed mood.

In addition to decreases in 5-HT and DA, we found a reduction in prefrontal cortical and hippocampal NA in the FH/HamSlc rats under basal conditions (Table 3), which is also known to contribute to the pathophysiology of depression (Köhler et al., 2016; Savitz and Drevets, 2013). In addition, the FH/HamSlc rats exhibited a decreasing tendency in the levels of the three monoamines, especially in the prefrontal cortex which has been shown to regulate mood and emotion via coordination of subcortical regions, such as the amygdala and hypothalamus (Drevets, 2000). Low 5-HT and NA concentrations may cause mood dysregulation by reducing prefrontal cortex functions. In addition, monoamines in the prefrontal cortex and hippocampus also play a role in memory performance, learning, and cognition (Bekinschtein et al., 2013; Mello-Carpes et al., 2016; Ramos and Arnsten, 2007; Reichel et al., 2012). Although the reduced concentrations of monoamines are expected to cause abnormal depression-like behaviours and cognitive dysfunction in FH/HamSlc rats, our behavioural analyses did not reveal any depression-like behaviours and cognitive dysfunction under basal conditions (Figs. 1,2,3, and 4A). One reason for this may be that the monoaminergic systems might undergo functional compensation through alterations in the number and/or sensitisation of receptors and transporters. FH rats show alterations in responses to serotoninergic agents, indicating altered 5-HT receptor sensitivity in the central nervous system (Gudelsky et al., 1985; Joseph, 1978). As for the absence of cognitive abnormalities, another reason may be that this test takes advantage of the rats’ innate behaviour and therefore represents a task with low cognitive load. Thus, monoaminergic abnormalities may not have been sufficient to inhibit the rats’ successful test performance under non-stress basal conditions, suggesting that FH/HamSlc rats do not have serious cognitive dysfunctions. Regarding the mechanism of bipolar disorder associated with receptor sensitivity, Berk et al. (2007) proposed a dopamine hypothesis for bipolar disorder stating that dopaminergic function alteration and the subsequent secondary changes in dopamine receptor sensitivity are involved in mood swings between manic and depressive phases. We observed stimulus-dependent behavioural changes that may be related to this mechanism, although not corresponding to mood swings in bipolar disorder. Although there are some differences between brain regions, it has been shown that the forced swim test facilitates monoamine release (Browne et al., 2014; Renard et al., 2003). We think that the transient suppression of hyperactivity after the forced swim task in FH/HamSlc rats is due to the overlap of the changes in receptor sensitivity and the enhancement of monoamine release. Taking the behavioural and neurobiological results together, this study shows that FH/HamSlc rats exhibit mania-related behavioural and monoaminergic abnormalities, although the relationship between behavioural and monoaminergic abnormalities is not fully clear.

The present results highlight the developmental differences between FH/HamSlc and F344 rats. The FH/HamSlc rats had a higher body weight than the F344 rats; however, their brain weight (and consequent brain/body ratio) was lower (Table 1). Incidentally, in a previous study, FH/HamSlc rats (274 ± 6 g) had a body weight similar to that of FH/Har rats (287 ± 6 g), but not that of FH/Wjd rats (384 ± 7 g), at 10 weeks (Overstreet and Rezvani, 1996). The behavioural differences observed in the present study could have been possibly caused by developmental differences in body and brain weights. However, we believe that these behavioural differences are more likely based on monoaminergic dysregulation. First, while there are developmental differences, no studies have reported that FH rats have anatomical and morphological abnormalities in the central nervous system, which would make cognitive dysfunction more likely. Second, the spontaneous alternation test did not reveal any differences in cognitive performance between the FH/HamSlc and F344 rats in the present study (Fig. 3A). Third, although body weight could affect moving speed and distance, we found slightly higher moving speed in the FH/HamSlc rats (Fig. 1C). However, the hyperactivity observed was mostly due to movement duration rather than speed (Fig. 1D). This suggests that body weight differences are unrelated to the behavioural abnormalities in this study. Finally, compared with F344 rats, the FH/HamSlc rats showed differences in monoaminergic dynamics, which are commonly associated with the altered behaviours observed in this study. In contrast to body weight, the FH/HamSlc rats exhibited a lower brain weight than the F344 rats (Table 1). This is concordant with a previous study that reported a reduced brain volume in FH rats compared to Sprague-Dawley rats (Joseph, 1978). Neural loss or brain atrophy is also often observed in patients with depression and bipolar disorder (Bora et al., 2010; Selvaraj et al., 2012). Although not equivalent to brain atrophy, the reduced brain size in the FH/HamSlc rats may indicate a reduced number and/or size of neurons and/or glial cells compared with F344 rats, which may affect the concentrations of monoamines and induce the mania-like characteristics and behaviours in the FH/HamSlc rats.

The present results suggest that FH/HamSlc rats can serve as an animal model for mania including bipolar disorder. Currently, several animal models of bipolar disorder exist; however, models sufficiently exhibiting both mania- and depression-related features are rare (Beyer and Freund, 2017). Furthermore, most animal models of bipolar disorder are also used for other psychiatric disorders, such as schizophrenia and ADHD (Beyer and Freund, 2017; van Enkhuizen et al., 2014a, b; Zhuang et al., 2001), as these animals also show impairments in attention or cognition. However, the FH/HamSlc rats in this study exhibited no evidence of severe abnormalities in attention or cognitive function, and they appear to be different from the schizophrenia and ADHD models. Therefore, we argue that FH/HamSlc rats are animal models of mania including bipolar disorder in terms of behavioural and monoaminergic abnormalities. Bipolar disorder has underlying genetic causes and is thought to develop via a combination of risk genes (Efimova et al., 2016); however, the responsible genes have not yet been identified. FH rats have a mutation in the recessive red-eyed dilution gene on chromosome 1. However, the loci associated with brain function have not yet been identified. Different FH rat strains show distinct behavioural features, indicating that these mutations may not directly affect behaviours; therefore, the differences among FH strains may be caused by a combination of other gene variants. FH/HamSlc rats are likely to have several gene variants that are responsible for bipolar disorder-like features and could, therefore, be considered as an animal model with a pathogenesis similar to that of bipolar disorder.

Previous findings on the usefulness of FH rat strains as psychiatric models are controversial (Overstreet and Rezvani, 1996). The present study, interestingly, demonstrates that FH/HamSlc rats have mania-like features which are different from those of other FH strains that exhibit depression- or anxiety-like behaviours. Behavioural analyses demonstrated hyperactivity and impulsivity under basal conditions, which indicate mania-related features. In addition, this hyperactivity was suppressed immediately after the forced swim test, which is similar to the clinical finding that exercise relieves the symptoms of bipolar disorder. These mania-related behaviours may be associated with abnormalities of the subcortical region system due to dysregulation of the prefrontal cortex caused by monoaminergic abnormalities such as monoamine imbalance and serotonin and dopamine turnover enhancement. Additionally, the FH/HamSlc rats did not exhibit any severe impairments related to schizophrenia or ADHD. Further studies are required to validate this animal model for mania including bipolar disorder. First, behavioural and neurobiological investigations on stimulus reactivity such as changes in cognitive functions, neurotransmitters, receptors, and stress hormones, which have not been clarified in this study, will be needed. Second, it will also be necessary to investigate other behavioural and biological characteristics found in animal models of mania including bipolar disorder (Beyer and Freund, 2017). These studies might reveal features other than mania-like features, such as depression-like features. Third, we did not determine the predictive validity of FH/HamSlc rats, i.e. their effectiveness in the assessment of a therapeutic drug effect on behavioural and neurochemical abnormalities. Taken together, our study reveals that FH/HamSlc rats have both behavioural and neurobiological abnormalities related to mania. Therefore, our results suggest that FH/HamSlc rats have the potential to be used as a new animal model for mania including bipolar disorder.

## Conflicts of interest

All animals in this study were provided without charge from Japan SLC, Inc.

## Author contributions

Conceived and designed the experiments: AH. Analysed the data: AH and HK. Wrote the first draft of the manuscript: AH. Contributed to the writing of the manuscript: AH and YT. Agreed with manuscript results and conclusions: AH, HK, and YT. Jointly developed the structure and arguments for the paper: AH and YT. Made critical revisions: AH and YT. All authors reviewed and approved the final manuscript.

## Funding

This research did not receive any specific grant from funding agencies in the public, commercial, or not-for-profit sectors.
